# Spinal Cord Injury Causes Marked Tissue Rearrangement in the Urethra—Experimental Study in the Rat

**DOI:** 10.3390/ijms232415951

**Published:** 2022-12-15

**Authors:** Ana Ferreira, Sílvia Sousa Chambel, António Avelino, Célia Duarte Cruz

**Affiliations:** 1 Experimental Biology Unit, Department of Biomedicine, Faculty of Medicine of Porto, University of Porto, 4200-319 Porto, Portugal; 2 Translational Neurourology, Instituto de Investigação e Inovação em Saúde-i3S, IBMC, University of Porto, 4200-135 Porto, Portugal

**Keywords:** epithelium, nerve fibres, micturition, spinal cord injury, urethra, urethral sphincter

## Abstract

Traumatic spinal cord injury (SCI) results in the time-dependent development of urinary impairment due to neurogenic detrusor overactivity (NDO) and detrusor-sphincter-dyssynergia (DSD). This is known to be accompanied by massive changes in the bladder wall. It is presently less clear if the urethra wall also undergoes remodelling. To investigate this issue, female rats were submitted to complete spinal transection at the T8/T9 level and left to recover for 1 week and 4 weeks. To confirm the presence of SCI-induced NDO, bladder function was assessed by cystometry under urethane anesthesia before euthanasia. Spinal intact animals were used as controls. Urethras were collected and processed for further analysis. Following thoracic SCI, time-dependent changes in the urethra wall were observed. Histological assessment revealed marked urethral epithelium reorganization in response to SCI, as evidenced by an increase in epithelial thickness. At the muscular layer, SCI resulted in strong atrophy of the smooth muscle present in the urethral sphincter. Innervation was also affected, as evidenced by a pronounced decrease in the expression of markers of general innervation, particularly those present in sensory and sympathetic nerve fibres. The present data show an evident impact of SCI on the urethra, with significant histological rearrangement, accompanied by sensory and sympathetic denervation. It is likely that these changes will affect urethral function and contribute to SCI-induced urinary dysfunction, and they deserve further investigation.

## 1. Introduction

Micturition, defined as the coordinated process of urine storage by the urinary bladder and its periodic elimination by the urethra, is mediated by complex neuronal mechanisms involving central and peripheral neurons. The complexity of the neuronal mechanisms controlling lower urinary tract (LUT) function renders them vulnerable to injuries affecting the central nervous system, such as spinal cord injuries (SCIs) [1,2]. Traumatic SCIs are typically followed by a period of little or no bladder activity during the acute phase of the disease [3,4], resulting from the interruption of ascending and descending circuits responsible for regulating LUT function. After injury, several mechanisms are set in motion, and cells are recruited to promote the sealing and healing of the injury site [5,6]. With time, neuroplastic mechanisms operating at the lumbosacral spinal cord lead to the emergence of an alternative reflex pathway responsible for NDO [7,8]. NDO is characterized by strong and frequent involuntary bladder contractions and often courses with detrusor-sphincter-dyssynergia (DSD: a lack of coordination between the bladder and urethral sphincter contraction, causing urinary incontinence episodes) [1]. Despite the reduction in mortality and SCI-associated morbidities, reflecting the development of better pharmacological and surgical interventions, there is no efficient treatment to fully overcome SCI-induced incontinence symptoms. Available treatments aim to protect kidney function, reduce urinary tract infections, and promote continence. Typically, antimuscarinic drugs are initiated to reduce bladder overactivity and intravesical pressures [9,10], and this is often combined with intermittent self-catheterization. For refractory cases, intradetrusor injections of botulinum toxin remain the gold standard therapy [11,12]. Nonetheless, all these therapies are accompanied by bothersome side effects and may lose efficiency over time, leading to treatment discontinuation. Therefore, a breakthrough is urgently needed. New therapeutic strategies are currently being developed, some without a direct focus on the LUT [13,14], and others are being used to control bladder function in spinal intact patients with LUT dysfunction [15], but the effects on SCI-induced urinary impairment have yet to be investigated.

The development of NDO and DSD is accompanied by considerable post-SCI tissue reorganization in the bladder wall. It is known that detrusor muscle cells become hypertrophic, with thick bundles of connective tissue coursing between muscle fibers [16,17]. Changes in bladder innervation have also been described, including the impairment of parasympathetic fibres and the upregulation of sensory innervation following SCI [16,18]. In contrast, the consequences of SCI in tissue organization within the urethral wall have been less well addressed. The urethra is a tubular organ, traditionally seen as the mere outlet for urine removal. In the proximal urethra, i.e., closer to the urinary bladder, the mucosa is lined with an epithelium with transitional properties, gradually changing to a stratified lining epithelium towards the distal urethra. Beneath the lamina propria, the urethral sphincter consists of two muscular layers. The inner layer, the internal urethral sphincter (IUS), which is composed of smooth muscle fibres extending from the bladder, is under the control of the autonomic nervous system. The outer muscular layer, the external urethral sphincter (EUS), controlled by the somatic nervous system, is composed of striated muscle cells and is responsible for voluntary sphincter closure during storage.

Accumulating evidence indicates that the urethra plays a role in LUT function rather than simply serving as a conduit for urine expulsion. In humans and animal models, different protocols of electrical stimulation of the urethra alter bladder activity [19,20]. In awake ewes and humans, urine flow along the urethra facilities bladder contractions, while urethral anesthesia impairs bladder emptying [21,22]. More recently, the presence of specialized epithelial cells, which release peripheral neurotransmitters involved in urethra–vesical crosstalk, has also been demonstrated [23,24]. Although undescribed, it is likely that the urethra might be affected by traumatic SCI, contributing to the impairment of LUT function. Thus, the present study sought to investigate time-dependent changes in the tissue organization of the urethral wall and in nerve fibers coursing in the mucosa and sphincter.

## 2. Results

### 2.1. Complete Spinal Cord Transection Alters Bladder Reflex Activity

**Figure 1:**
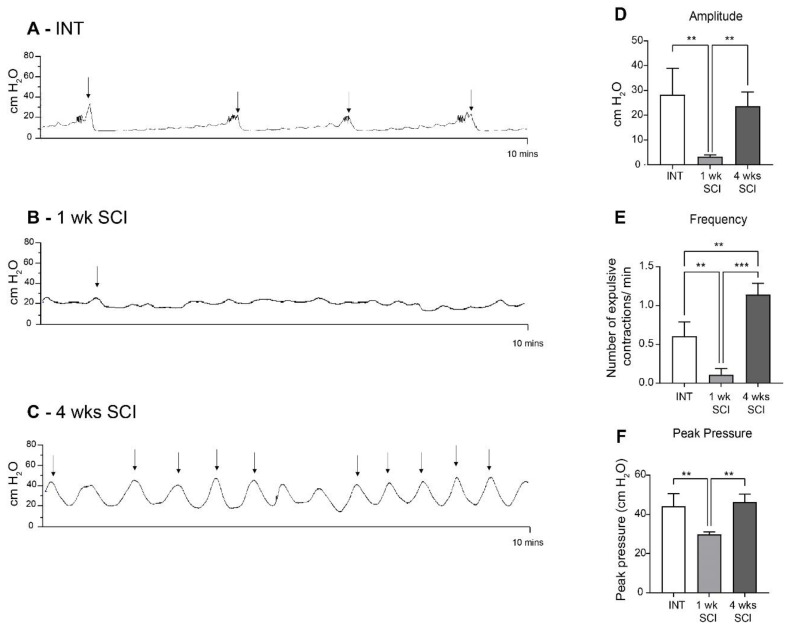
Bladder reflex activity. Bladder contractility was assessed by cystometry under urethane anesthesia. Intact animals (**A**) presented a typical bladder function. One week post-SCI (**B**), SCI animals were considered to be in spinal shock, with an almost total absence of bladder contractions. In 4-week SCI rats (**C**), bladder contractions were partially restored, with a pattern of bladder hyperreflexia. Urodynamic parameters, including the amplitude (**D**), frequency (**E**), and peak pressure (**F**) of the expulsion bladder contractions of the overall experimental groups (*n* = 4). One-way ANOVA followed by Tukey’s multiple comparison test (** *p* < 0.01; *** *p* < 0.001;). The arrows indicate the expulsion of a drop of urine.

Before tissue collection, all animals were submitted to cystometries under urethane anesthesia. Spinal intact (INT) animals presented normal bladder function (Figure 1A,D–F; Table 1). One week after the spinal lesion, bladder reflex contractions were almost abolished (Figure 1B,D–F; Table 1). At 4 weeks post-SCI, it was possible to observe frequent and high-amplitude bladder reflex contractions, with a typical pattern of detrusor hyperactivity (Figure 1C–F; Table 1).

### 2.2. Spinal Cord Transection Affects the Organization of the Urethral Epithelium

**Figure 2:**
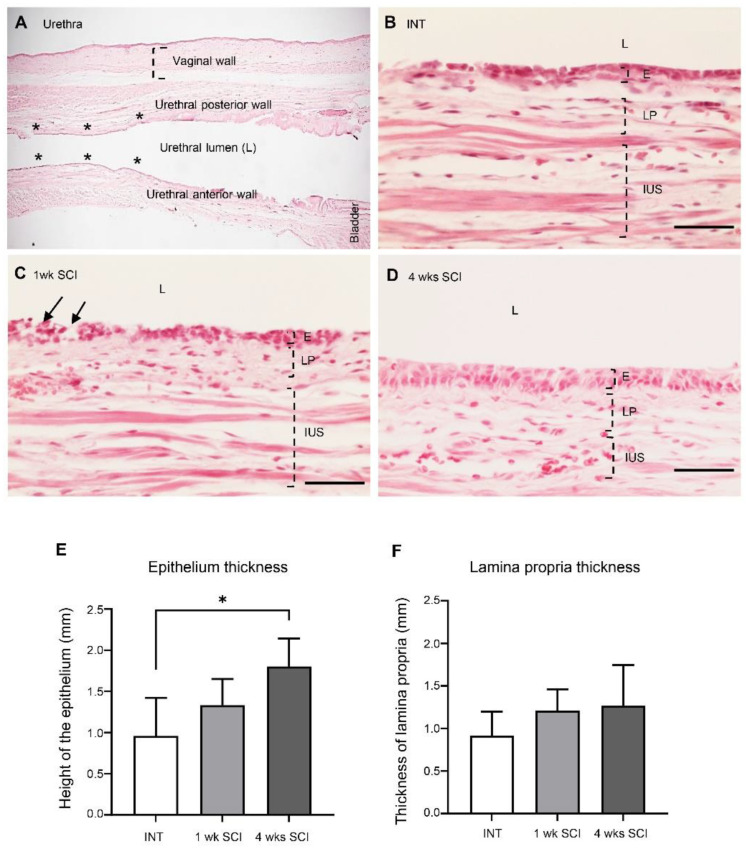
Epithelium and lamina propria organization. The morphology of the urethral wall was studied using hematoxylin and eosin-stained urethral sections. A general view of the urethra is represented in (**A**). The localizations of the vaginal and the urethral wall (posterior and anterior), the urethral lumen, and the bladder are identified. The six locals chosen for measures are marked as *. Intact animals (**B**) presented a well-organized epithelium (**E**) and lamina propria (LP). One-week (1 wk SCI) spinal injured rats (**C**) presented severe damage in the mucosa, with some signs of cell desquamation (pointed by arrows) in the E and some bleeding of the LP (not shown). Four weeks (4 wks SCI) after the spinal insult (**D**), the E and LP recovered its organization. Scale bars equal 50 μm. Quantification by Image J of the height of the epithelium (**E**), showing a significant increase in 4-weeks SCI animals. No changes were found in the thickness of the lamina propria (**F**) (*n* = 4). One-way ANOVA followed by Tukey’s comparison test (* *p* < 0.05).

To assess the consequences of SCI in the tissue organization of the urethral wall, longitudinal urethral sections were stained with haematoxylin and eosin. Measurements of the thickness of each histological layer were performed where indicated (Figure 2A). Urethral sections from spinal intact animals showed evidence of a stratified epithelium, with several layers, the most superficial of which was composed of flattened cells (Figure 2B). The epithelium thickness was 0.96 ± 0.47 µm (Figure 2E). One week after spinal injury, epithelial disorganization was evident, with signs of cellular loss in the most superficial layers (Figure 2C). The thickness of the epithelium was 1.3 ± 0.32 µm (Figure 2E). Four weeks after spinal lesion, epithelial layers were again evident, with fewer signs of cellular loss and a higher number of superficial flattened cells (Figure 2D). The epithelial height was 1.80 ± 0.34 µm (Figure 2E; *p* < 0.05 vs. INT). The thickness of the lamina propria remained unaltered (Figure 2F).

### 2.3. Changes in the Urethral Sphincter after SCI

#### 2.3.1. Internal Urethral Sphincter

**Figure 3:**
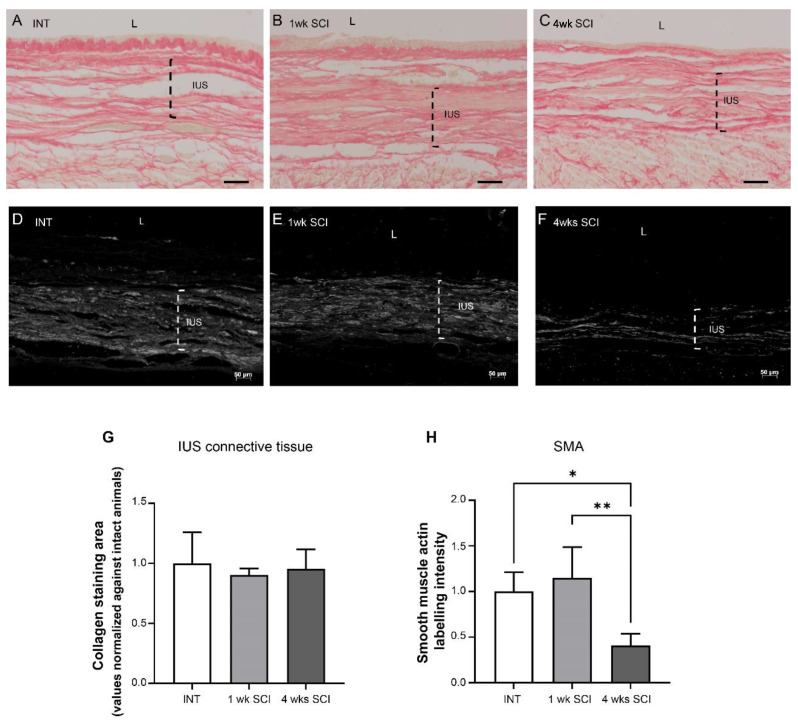
Internal urethral sphincter integrity. The integrity of the internal urethral sphincter (IUS) was measured by Sirius Red staining. Intact (**A**), 1 wk SCI (**B**) and 4 wks SCI (**C**) animals presented no changes in connective tissue arrangement (**G**). Immunostaining of smooth-muscle actin (SMA) showed that spinal intact (**D**) and 1-week SCI animals (**E**) presented a tick IUS with strong actin expression. In sections from animals left to recover 4 weeks after spinal lesion (**F**), there was evidence of a marked decrease in SMA expression. The quantification of the IUS labelling intensity of actin in the proximal urethra (**H**) (*n* = 4). One-way ANOVA followed by a Tukey’s multiple comparison test (* *p* < 0.05 versus INT; ** *p* < 0.01 versus 1-week SCI).

Paraffin-embedded urethral sections were stained with Sirius Red to investigate the collagen content in the urethral wall (Figure 3A–C,G). In the IUS, Sirius red staining showed no differences in stained connective tissue, with bundles of connective fibers, most likely collagen, coursing between smooth muscle fibers (Figure 3A–C,G).

The expression of smooth muscle actin (SMA) was also analyzed in paraffin-embedded urethral sections. The abundant expression of SMA was found in INT animals (Figure 3D,H), and similar observations were made in sections obtained from 1-week post-SCI animals (Figure 3E,H). In contrast, SMA expression was significantly reduced in sections from 4-weeks post-SCI animals (Figure 3F,H; *p* < 0.05 vs. INT and *p* < 0.01 vs. 1 week post-SCI).

#### 2.3.2. External Urethral Sphincter

**Figure 4:**
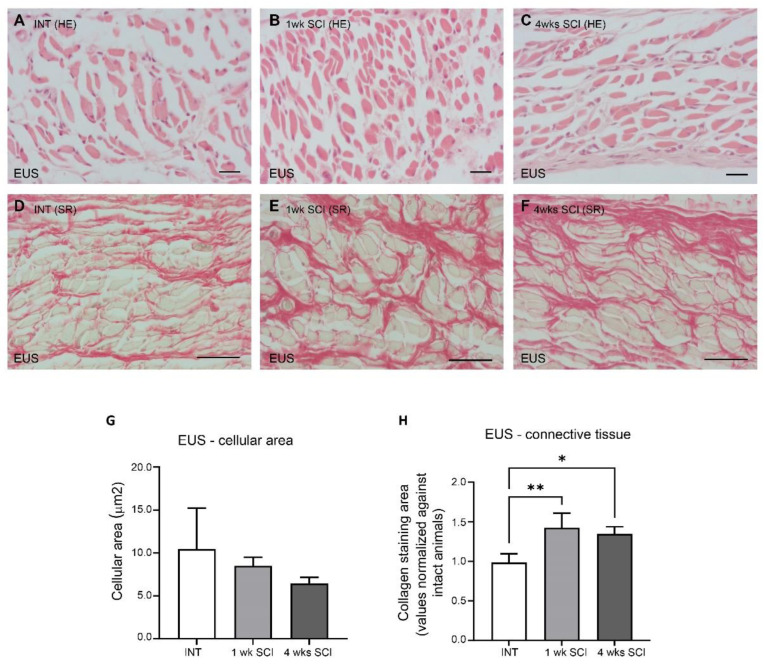
External urethral sphincter integrity. The morphology of the external urethral sphincter (EUS) striated cells was assessed by staining paraffin-embedded sections with haematoxylin–eosin. Spinal intact animals (**A**) presented long and slender striated cells, as well as 1-week (**B**) and 4-weeks SCI animals (**C**), with no changes in the cellular area (**G**). The levels of fibrosis, estimated by the collagen content stained with Sirius Red, significantly increased in SCI animals at 1 week post-SCI (**E**) and were still evident at 4 weeks after spinal lesion (**F**) when compared to controls (**D**,**H**) (* *p* < 0.05; ** *p* < 0.01). Scale bars equal 50 μm. One-way ANOVA followed by a Tukey’s multiple comparison test.

The effects of SCI on the arrangement of the external urethral sphincter were also analysed. In INT sections stained with haematoxylin–eosin, it was possible to observe long striated muscle fibers (Figure 4A). The average transverse area of striated fibres was 10.48 ± 4.8 μm^2^ (Figure 4G). Observations were also made in sections from 1-week and 4-weeks post-SCI animals (Figure 4B,C), and the average transverse areas, respectively, were 8.52 ± 1.00 and 6.43 ± 0.73 μm^2^ (Figure 4G), suggesting a trend towards slimmer muscle fibres that did not reach statistical significance. Sirius red staining showed significant signs of fibrosis in SCI animals, with a thick bundle of connective fibres between striated muscle cells evident at 1 and 4 weeks post-SCI (Figure 4D–F,H; * *p* < 0.05 and ** *p* < 0.01 versus INT).

### 2.4. Urethral Innervation after SCI

#### 2.4.1. General Innervation

**Figure 5:**
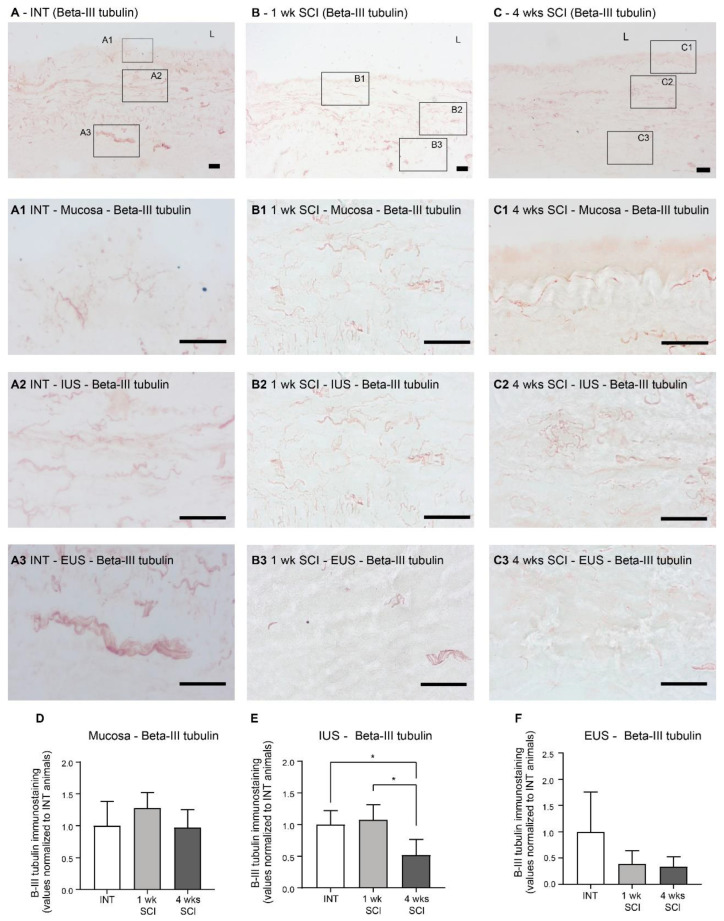
Immunolabeling of β-III tubulin in the proximal urethra. The general neuronal content was assessed using the ABC method for the detection of β-III tubulin, which stained the overall population of neuronal fibers in the urethral wall. Spinal intact animals (**A**) presented fibers widely distributed along the wall, in the mucosa (**A1**), in the internal urethral sphincter (IUS) (**A2**), and in the external urethral sphincter (EUS; (**A3**)). One-week SCI animals (**B**) presented an evident denervation in the EUS (**B3**) but not in the mucosa (**B1**) and IUS (**B2**). Animal lefts to recover 4-weeks post-SCI (**C**) presented denervation both in IUS (**C2**) and EUS (**C3**) but not in the mucosa (**C1**). Scale bars equal 50 μm. The quantification of labeling intensity by Image J in the mucosa (**D**), IUS (**E**), and EUS (**F**) (*n* = 5). One-way ANOVA followed by Tukey’s multiple comparison test (* *p* < 0.05 versus spinal intact animals).

Changes in urethral innervation associated with SCI were investigated by immunostaining urethral sections from INT and SCI rats. General innervation was evaluated by the expression of β-III tubulin, a pan-neuronal marker (Figure 5A–C). In the mucosa, no significant differences in β-III tubulin expression were found (Figure 5A–C,A1–C1,D). In contrast, a reduction was found in the urethral sphincter. In the IUS, while β-III tubulin remained unaltered at 1 week post-SCI (Figure 5B,B2,E) in comparison with INT (Figure 5A,A2,E), a significant decrease was observed 4 weeks following the spinal lesion when compared to INT and 1-week post-SCI animals (Figure 5C,C2,E; *p* < 0.05 vs. INT and 1w SCI). In the EUS, values did not reach statistical significance, although a tendency for early denervation was already observable at 1 week following the spinal lesion (Figure 5B,B3,C,C3,F).

#### 2.4.2. Sensory Innervation

**Figure 6:**
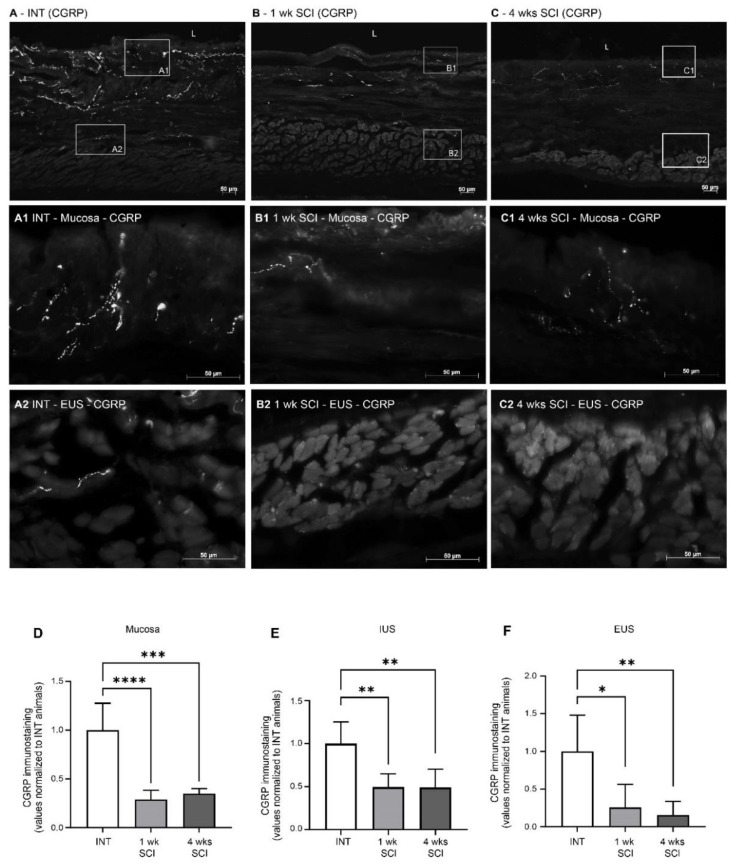
Distribution of CGRP-positive fibres in the proximal urethra. Sensory innervation was assessed by the immunostaining of the CGRP (calcitonin gene-related peptide) present in sensory peptidergic fibres. Intact animals (**A**) presented peptidergic fibres in the mucosa (**A1**), and some penetrated the epithelium (**E**) and through the lamina propria (LP). Some CGRP-positive fibres were also present in the external urethral sphincter (EUS) (**A2**). One- and four-weeks SCI animals ((**B**,**C**), respectively) presented a marked decrease in CGRP expression in the mucosa (**B1**,**C1**) and in the EUS area (**B2**,**C2**). Scale bars equal 50 μm. Quantification of labelling intensity by Image J in the mucosa (**D**), IUS (**E**), and EUS ((**F**) *n* = 5). One-way ANOVA followed by Tukey’s multiple comparison test (**** *p* < 0.001; *** *p* < 0.0001, ** *p* < 0.01; * *p* < 0.05 versus spinal intact animals).

As β-III tubulin levels suggested the occurrence of the denervation of the urethral wall, the effects of SCI on the sensory fibres present were assessed by evaluating calcitonin gene-related peptide (CGRP) immunostaining. In sections from spinal intact animals, CGRP-positive fibres were present predominantly in the mucosa, with fibres penetrating the epithelial layer and coursing in the lamina propria (Figure 6A,A1). After SCI, CGRP expression in the mucosa was significantly decreased at both time points after the spinal lesion (Figure 6B,B1,C,C1,D; *p* < 0.0001 vs. INT for both time points). In the muscular layer of INT animals, CGRP expression was less abundant (Figure 6A). As in the mucosa, CGRP levels in the IUS were also decreased after SCI (Figure 6B,C,E *p* < 0.01 vs. INT for both time points). This reduction was also evident in the EUS (Figure 5B2,B3,F; *p* < 0.01 vs. INT for both time points).

#### 2.4.3. Sympathetic Innervation

**Figure 7:**
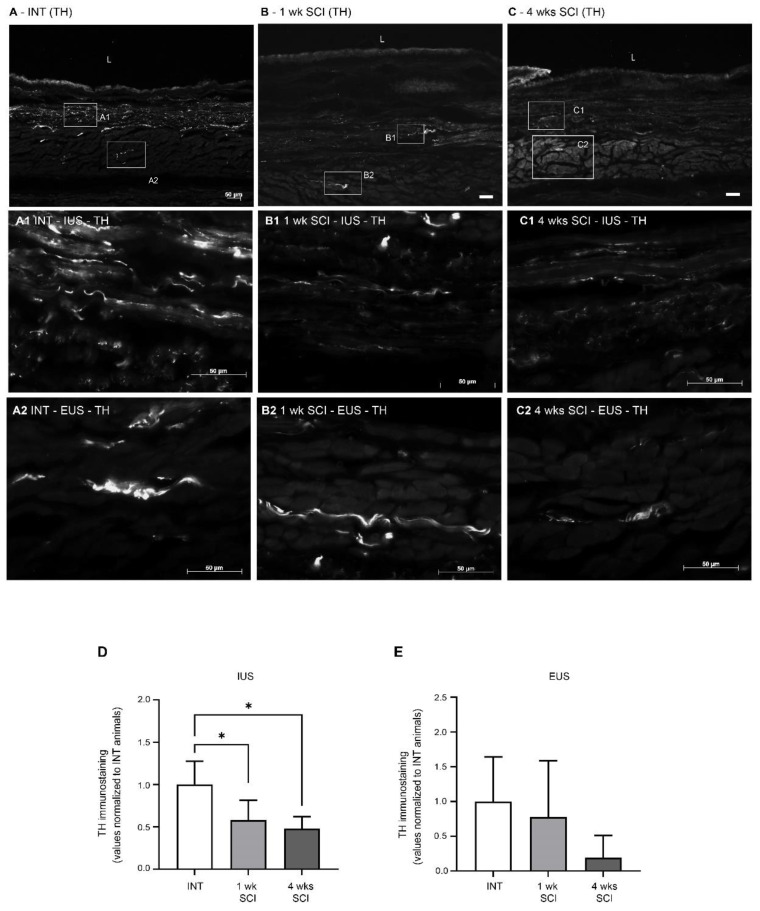
Expression of tyrosine hydroxylase (TH) in the urethral sphincter. Sympathetic innervation was analysed using TH immunohistochemistry, a noradrenergic marker for sympathetic fibres. Intact animals (**A**) presented TH-positive fibres predominantly in the internal and external urethral sphincter (IUS, (**A1**); EUS, (**A2**), respectively). No TH fibres were observed in the mucosa. Positive fibres were also present in sections from 1-week SCI animals (**B**,**B1**,**B2**), in which a prominent reduction was observed. This sympathetic denervation was also evident in sections from 4-weeks post-SCI animals (**C**), both in the IUS (**C1**) and EUS (**C2**). Scale bars equal 50 μm. The quantification of labelling intensity by Image J in the IUS (**D**) and EUS (**E**) (*n* = 4). One-way ANOVA followed by Tukey’s multiple comparison test (* *p* < 0.05 vs. INT).

Sympathetic innervation was evaluated by tyrosine hydroxylase (TH) immunostaining. In intact animals, no TH-positive fibres were found in the mucosa (Figure 7A), and immunoreactive fibres were predominantly distributed in the IUS (Figure 7A1). Some positive profiles were also present in the EUS (Figure 7A2). One week and four weeks after the spinal lesion, TH immunoreactive fibres in the IUS were significantly reduced (Figure 7B1,C1,D; *p* < 0.05 vs. INT). In the EUS, no differences were found between the experimental groups (Figure 7A2–C2,E).

#### 2.4.4. Parasympathetic Innervation

**Figure 8:**
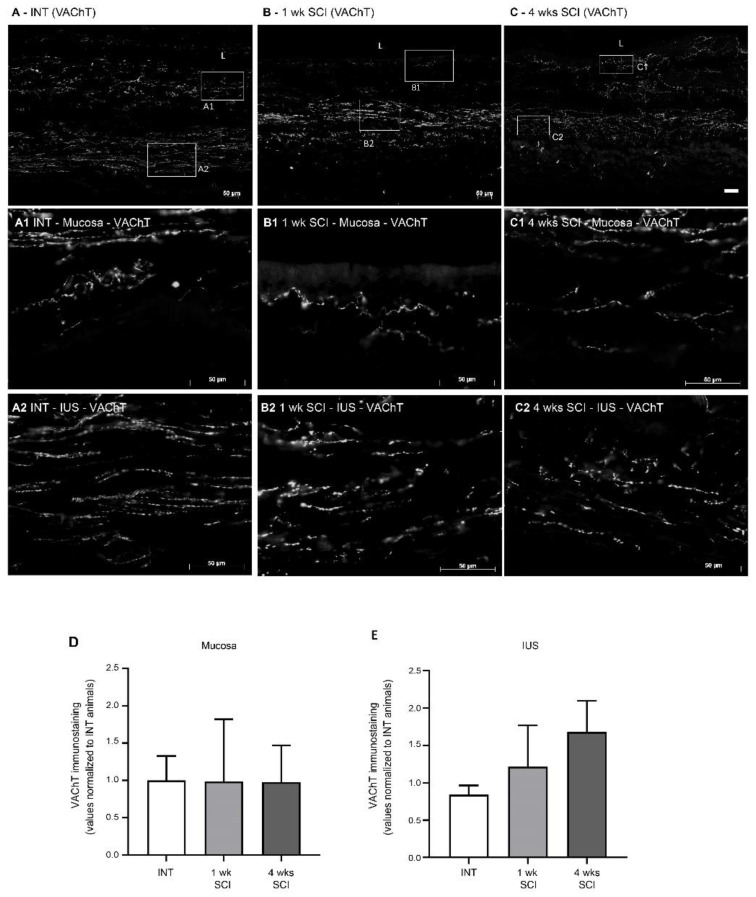
Immunolabeling of VAChT in the proximal urethra. Parasympathetic innervation was assessed by immunohistochemistry against the Vesicular Acetylcholine Transporter (VAChT), which stains cholinergic fibres. Intact animals (**A**) presented sympathetic fibres in the mucosa, penetrating the epithelial layer (**E**) and expanding in the lamina propria (LP) (**A1**). Immunoreactive fibres were also present in the internal urethral sphincter (IUS) (**A2**). A similar pattern was found in 1-week post-SCI animals (**B**,**B1**,**B2**). In sections from 4-weeks post-SCI animals, there was a trend towards an increase in VAChT immunoreactivity (**C**) in the mucosa and internal urethral sphincters ((**C1**); IUS, (**C2**), respectively). No VAChT fibres were observed in the EUS. Scale bars equal 50 μm. No significant changes were found in the mucosa (**D**) or IUS (**E**). The quantification of labelling intensity by Image J in the extension of the proximal urethra (*n* = 4). One-way ANOVA followed by Tukey’s multiple comparison test.

Parasympathetic innervation in the urethra was assessed by immunostaining against VAChT. In intact animals, parasympathetic tracts were observed mostly at the mucosa (Figure 8A,A1) and in the IUS (Figure 8A,A2), with no signs of VAChT-positive fibres in the EUS. After SCI, no significant changes were observed in the mucosa (Figure 8D). In the IUS, there was a trend for an increased intensity of VAChT immunostaining that did not reach statistical significance (Figure 8E), with abundant positive profiles observed in the mucosa and in the IUS at 1 (Figure 8B,B1,B2) and 4 weeks post-SCI (Figure 8C,C1,C2).

#### 2.4.5. Sprouting of Nerve Fibers

**Figure 9:**
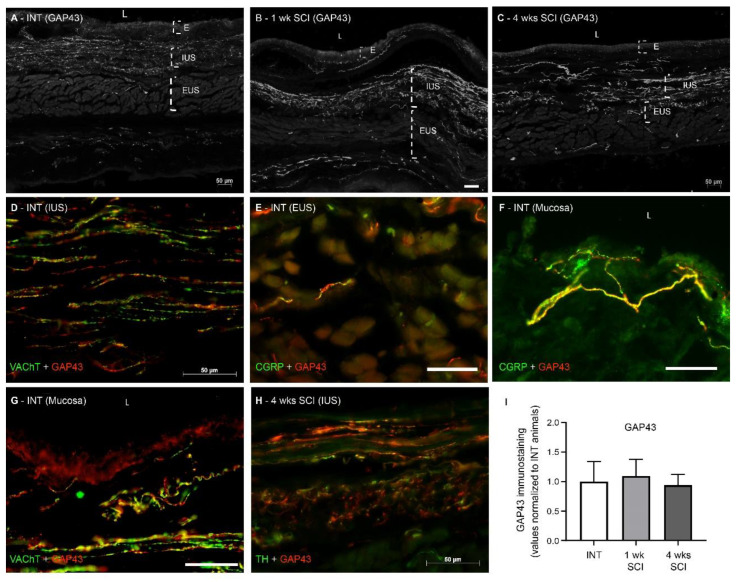
Immunolabeling of GAP-43 in the proximal urethra. The existence of neuronal sprouting was assessed by immunohistochemistry against Anti-Growth Associated Protein-43 (GAP-43), which stains sprouting axons. GAP43 was abundantly expressed in urethral sections from intact (**A**), 1-week (**B**) and 4-weeks post-SCI animals (**C**). The co-localization of GAP-43 with all markers of nerve fibres was observed (**D**–**H**), irrespective of the time point of disease progression and location within the urethral wall. We found no significant differences in GAP-43 content (**I**), irrespective of the time point of disease progression. Scale bars equal 50 μm. The quantification of labelling intensity by Image J in the extension of the proximal urethra (*n* = 4). One-way ANOVA followed by Tukey’s multiple comparison test.

As axonal sprouting in the bladder accompanies NDO emergence [25,26], GAP-43 expression in the urethra was assessed. We found no significant differences between the experimental groups (Figure 9A–C,I) and observed the co-localization of GAP-43 with all markers of nerve fibres (Figure 9D–H), irrespective of the time point of disease progression.

## 3. Discussion

The present study investigated tissue reorganization in the urethra associated with urinary dysfunction caused by SCI. We observed profound histological changes, including a marked atrophy of the IUS. In addition, we also found evidence of a loss of sensory and sympathetic fibres of the urethral wall, possibly contributing to SCI-induced urinary impairment. To our knowledge, this study provides the first evidence of time-dependent histologic and innervations changes taking place in the urethra wall following SCI.

Here, a transection-type rat model of SCI was chosen, and a complete spinal transection at the T8-T9 level was performed. LUT function was analysed under urethane anaesthesia. As before [26], bladder reflex activity was strongly reduced 1 week after SCI, consistent with spinal shock [4]. Four weeks post-SCI, the frequency and amplitude of bladder contractions was markedly increased, indicating that NDO was already established. An analysis of haematoxylin/eosin-stained urethral sections showed that spinal intact animals presented a stratified epithelium. One week post-SCI, a marked disorganization was observed in the epithelium lining the proximal urethra, with signs of desquamation in the superficial layers. These results are in line with studies performed in bladders from SCI rats in acute stages of the disease, reporting alterations in epithelial morphology, including a loss of apical cells, general disorganization, and a reduction in cellular volume [17,27]. At 4 weeks post-SCI, urethral epithelial layers were again evident, with fewer signs of cellular loss and a higher number of superficial flattened cells. The epithelium height had significantly increased when compared with intact animals. These changes are similar to those observed in bladder sections obtained from SCI animals at the same time point. Indeed, 4 weeks after SCI, the bladder epithelium had recovered its organization, but the expression of differentiation markers indicated incomplete regeneration [27]. Although not explored in the present work, one could speculate about the occurrence of a similar process in the urethra. The reasons explaining the high cellular turnover in the LUT epithelium, including at the urethra, may result from the exposure to high levels of catecholamines, such as norepinephrine, released in the bladder following acute SCI [17] but likely not maintained in chronic stages of disease progression.

The urethral sphincter is the functional contractile unit of the urethra; it is responsible for the control of the bladder neck open and, therefore, urine expulsion. The internal layer of the sphincter, the IUS, is composed of smooth muscle fibres extending from the bladder body and controlled by the autonomic nervous system. The external layer of the sphincter comprises striated muscle fibres operating under the activity of the somatic nervous system. As in the urethral mucosa, changes in the urethral sphincter were also found, including signs of fibrosis in the EUS but not in the IUS. We also observed marked muscle atrophy of the IUS, as evidenced by a significant decreased expression of SMA at 4 weeks post-SCI. This is in contrast with observations performed in the bladder. Previous studies reported muscle hypertrophy [16] and changes in orientation of the detrusor smooth muscle fibres [28], accompanied by signs of fibrosis [16,29]. These morphological changes in the bladder may reflect adjustments linked to organ enlargement [28] and are considered part of the compensatory mechanisms operating to adjust this organ to the increased mechanical demands on the bladder wall due to prolonged periods of high intravesical pressure [28]. As the urethra is not the urine reservoir and thus does not suffer increases in pressure and distention during storage, it is possible that, unlike the detrusor, the IUS becomes atrophic and does not increase collagen content. Fibrosis was only seen in EUS, suggesting that the occurrence of tissue rearrangement likely does not affect the volume of striated muscle cells, as the transverse area of these cells was not altered by SCI.

The consequences of SCI in the urethral innervation were also assessed by analyzing the expression of the pan-neuronal marker β-III tubulin. We found signs of denervation, and by using specific antibodies, it was possible to identify a significant loss of sensory (mucosa, IUS, and EUS) and sympathetic (IUS) fibres, but no changes in parasympathetic innervation were found. While the distribution of peptidergic fibres in the bladder of intact and SCI individuals is well described [18], to our knowledge, the same does not apply to the urethra after SCI. Like others [30], in spinal intact animals, we identified a suburothelial sensory nerve plexus extending into the urethral epithelium. In the muscular layer, CGRP-positive afferents were also present in the IUS, with some CGRP-positive fibres also in the EUS. Therefore, the urethral sensory innervation follows a similar distribution as in the bladder, where CGRP-positive fibres were described in the urothelium [31], in the supporting lamina propria, and close to smooth muscle cells. Urethral CGRP expression was strongly impacted by SCI, with a marked decrease being observed at 1 week post-SCI in both the mucosa and muscular layer. This is in contrast with observations made in the bladder, as previous studies demonstrated the post-SCI sprouting of sensory afferents [26], which also occurred in the neuronal pathways regulating LUT function [25,32,33]. This impairment of the sensory innervation of the urethra may contribute to SCI-induced detrusor-sphincter dyssynergia. In mice, it has been demonstrated that the relaxation of the urethral sphincter during voiding is regulated by capsaicin-sensitive C-fibre bladder afferents [34]. While yet to be demonstrated, one could speculate that a reduction in the population of urethral peptidergic afferents, most likely sensitive to capsaicin, as in the bladder [31], should impair sphincter relaxation and contribute to the loss of coordination between detrusor contraction and the relaxation of the urethral sphincter.

Sympathetic innervation of the urethra was analysed by assessing the expression of TH. These fibres are critical in promoting urine storage, as they induce sphincter contraction via the release of norepinephrine while relaxing the bladder body [35]. In spinal intact animals, TH-positive fibres were predominantly observed in the IUS and were notably absent in the mucosa. SCI animals presented a marked decrease in sympathetic innervation, observed as early as 1-week post-lesion and maintained at 4 weeks post-SCI, when LUT function was clearly impaired. Together with the loss of urethral sensory innervation, the sympathetic denervation of the IUS could further contribute to aberrant sphincter activity and LUT dysfunction.

Interestingly, the lack of sympathetic input may help to explain IUS atrophy. One should remember that, in other systems, including blood vessels, it has been demonstrated that norepinephrine has a trophic effect on smooth muscle cells [36,37]. It is possible the same could happen in the urethra, where a lack of sympathetic input could have resulted in the IUS atrophy of smooth muscle cells. Importantly, smooth muscle cells are established sources of Nerve Growth Factor (NGF) in the LUT, releasing this neurotrophin upon stretching [38,39]. As peptidergic afferents, including those present in the urinary bladder, are highly dependent on NGF [40], one could hypothesize that a lack of sympathetic input in the urethra may lead to IUS atrophy, triggering a reduction in NGF tissue levels, which, in turn, could lead to a loss of CGRP-positive fibers due to NGF deprivation. A reduction in NGF could further accentuate sympathetic loss, as sympathetic neurons are also dependent on this neurotrophin for survival [41]. The reasons, however, for sympathetic denervation remain unclear, and it is difficult to pinpoint the initial trigger for this dysregulation.

In the bladder, parasympathetic innervation is particularly important in the voiding phase. During storage, these circuits are silent and are activated only when the bladder reaches its maximum capacity to promote detrusor contraction for urine expulsion [42]. In contrast, in the urethra, parasympathetic innervation has an inhibitory effect mediated by nitric oxide release, resulting in sphincter relaxation. Intact animals presented positive VAChT-positive fibres in suburothelial layers. In some cases, some parasympathetic fibres extended their processes into the urothelium, as previously observed by other investigators [30]. Parasympathetic fibres were also present in the IUS, but very few were observed in the EUS. The analysis of urethral sections obtained from SCI animals, irrespective of the time point, did not show any significant differences when compared to intact animals. This is in contrast with observations made in the bladder of SCI animals. In this case, the parasympathetic innervation of the detrusor was the most affected by SCI, with studies demonstrating that VAChT fibres decreased in the first days after SCI, partially recovering with the time course but failing to reach basal levels [43]. The reasons why urethral parasympathetic innervation was not affected by SCI remain elusive.

Because axonal expansion is known to be a key mechanism for NDO emergence [25,26,32], sprouting was also studied by assessing the expression of GAP-43, an established marker of axonal growth [44]. We found the co-localization of GAP-43 with all the nerve fibre markers studied, suggesting that all populations of nerve fibres coursing in the urethral wall undergo some degree of morphological plasticity. The quantification of GAP-43 expression showed no significant changes after SCI, contrasting to what is known to occur in the bladder and the neuronal pathways involved in micturition control [26,32]. While the sprouting of bladder afferents has been linked to high levels of NGF in the bladder [45], the concentration of NGF in the urethra wall should likely have decreased as a result of IUS atrophy, considering that smooth muscle cells are important sources of NGF [38,39]. Therefore, no abnormal axonal sprouting was observed in the urethra of SCI rats in comparison with spinal intact animals.

## 4. Materials and Methods

### 4.1. Animals and Drugs

The experiments were performed using in-house bred, 200–250 g Wistar rats, derived from the Charles River Laboratories (France) colony. Female rats were preferred considering the wealth of published data on urinary impairment after SCI using these animals. Moreover, female rats were also preferred because it is easier to accomplish urine removal during the bladder areflexia period. The animals were maintained under a 12 h light/dark schedule, with free access to food and water. Spinal cord injuries were performed under deep anesthesia induced by the intraperitoneal injection of ketamine (60 mg/kg) and medetomidine (0.25mg/kg). Anaesthesia was reverted with atipamezole (1 mg/kg), delivered as an intramuscular injection. For cystometries, the animals received a subcutaneous injection of urethane (1.2 g/kg), followed by sodium pentobarbital (400 mg/kg), prior to euthanasia. The antibodies used in the immunohistochemistry experiments are indicated in Table 1 and Table 2.

The experimental procedures were carried out according to the European Communities Council Directive 2010/63/EU and local regulations (protocol ORBEA_56_2017/1218; 31 January 2019). All efforts were made to reduce the number of animals used as well as to reduce animal stress and suffering.

### 4.2. Complete Spinal Cord Transection

After anesthesia and laminectomy between the T7-T10 vertebrae, the T8/T9 spinal segments were exposed for complete sectioning. A small piece of sterile haemostatic sponge was inserted between the retracted cord ends to limit bleeding. During the recovery period, the animals received oral antibiotics for 8 days (enrofloxacin 5 mg/kg), and, if necessary, they received analgesic drugs (tramadol 0.4 mg/kg). In order to avoid urinary retention, bladders were manually emptied daily by abdominal compression until automatic voiding was established. The experimental groups (*n* = 4–6 animals per group) included animals left to recover 1 (1 wk SCI) and 4 weeks (4 wks SCI) following the spinal lesion. Spinal intact rats (INT) were used as controls.

### 4.3. Cystometries and Terminal Handling

In order to evaluate the bladder reflex activity, the animals were submitted to cystometry under urethane anesthesia. The animals were placed in a heating plate to maintain body temperature at 37 °C. Following deep anesthesia, a suprapubic skin incision was made, and muscle bundles were separated for bladder exposure. A 21-gauge needle was inserted into the bladder dome, and sterile saline was infused for 1 h at a rate of 6 mL/h. Bladder contractions were recorded by a pression transducer connected to the needle. After cystometry, animals were euthanized with an intraperitoneal injection of sodium pentobarbital. Cystometrograms were analyzed using LabScribe software 2.0 (World Precision Instruments, Hertfordshire, UK).

### 4.4. Histological Analysis

After euthanasia, urethras were dissected, collected, and fixed overnight in 4% formalin solution. This was followed by sequential dehydration with different concentrations of ethanol and benzene before paraffin impregnation. Serial longitudinal 5 μm urethral sections were obtained in a Leica RM2145 microtome and collected in poly-L-lysine coated slides. For routine histological staining, sections were deparaffinised with benzene and gradually rehydrated with ethanol solutions. Haematoxylin–eosin staining was used to analyse tissue histology, and Sirius Red staining was used to assess collagen content as a measure of fibrosis levels. Images were obtained using a Carl Zeiss microscope equipped with a CH-9435 Leica camera. The Leica application suite (VAS 4.6.0) software was used for image capturing.

### 4.5. Immunohistochemical Tissue Assessment

Urethras were collected and fixed in 4% paraformaldehyde solution for 6 h before cryoprotection in 30% sucrose solution with 0.1% sodium azide for 24 h. Longitudinal urethral sections (14 μm thick) were obtained in a freezing cryostat, collected in Superfrost Plus slides, and stored at −20° until further processing.

The expression of β-III tubulin (a general marker of nerve fibres) was assessed using the ABC method for light microscopy. Briefly, after several washes with a phosphate-buffered saline solution (PBS), sections were incubated in 0.3% hydrogen peroxide solution in PBS to inhibit endogenous peroxidase. After subsequent washes, sections were blocked for 2 h with 10% normal horse serum in PBS containing 0.3% Triton X-100 (PBST) followed by a 48 h incubation at 4 °C with anti- β-III tubulin antibody. After several washes with PBST, sections were incubated with a biotinylated swine anti-rabbit antibody for 1 h. The immunoreaction was visualized using the ABC peroxidase-conjugated method (1:200; Vectorlabs, UK), using 3,3′-diaminobenzidine tetrahydrochloride (5 min in 0.05 M Tris buffer, pH 7.4, containing 0.05% DAB and 0.003% hydrogen peroxide) as chromogen. Sections were cleared in xylene, cover-slipped, and observed. All antibodies and the ABC complex were prepared in PBST.

Immunofluorescence techniques were used to analyse the expression of smooth muscle actin (SMA) and nerve fibre markers, including calcitonin gene-related peptide (CGRP), tyrosine hydroxylase (TH), and vesicular acetylcholine transporter (VAChT). To assess axonal sprouting in the urethra wall, an antibody against GAP43, a marker of axonal growth [44], was used in combination with the antibodies mentioned above. SMA levels were detected in paraffin-embedded sections, which were de-paraffinized, re-hydrated, and submitted to antigen retrieval using citrate buffer boiling.

In all cases, the slides were washed with PBST and blocked with 10% of normal horse serum (NHS) in PBST for 1 h at room temperature (RT). The sections were then incubated for 48 h at 4 °C with a primary antibody (Table 2), after which they were thoroughly washed and incubated for 1 h in a mixture of fluorescently labeled secondary antibodies (Table 3). Concentrations of primary and secondary antibodies are indicated in Table 2 and Table 3. Finally, sections were mounted using an anti-fade mounting medium (Thermo Fisher Scientific, Rockford, IL, USA) and observed in a fluorescence Zeiss microscope (Axioimager Z1, Zeiss Z1 from Zeiss) using the AxioVision 4.6 software.

### 4.6. Quantifications and Statistics

Cystometograms were analyzed using the LabScribe software (version 2.34900; iWorx Systems, Friedberg, Germany). Only bladder contractions with amplitudes superior to 10 cm H_2_O were quantified.

All image data were collected in the proximal urethra, both in the posterior (close to the vagina) and anterior side and quantified using the Image J software. The thickness of the urethral epithelium and lamina propria was measured as an average of six measurements per animal, as indicated in Figure 2A. Image J was also used to determine the collagen staining area and the area of EUS muscle fibres, measured in transversal individual sections. Labelling intensity was determined by densitometry and obtained in three regions of interest: mucosa, IUS, and EUS. In all cases, data are presented as an average of the values measured in both sides of the urethra ± standard deviation.

All statistical analyses were conducted using GraphPad 9.0 software. Datasets were first tested for normality (Shapiro–Wilk normality test), followed by variance analysis testing using one-way ANOVA followed by Tukey’s post hoc test. In all cases, *p* < 0.05 was considered statistically significant. The data are presented as the mean ± standard deviation (SD).

## 5. Conclusions

While many investigators have focused on the bladder, to our knowledge, this is the first study providing evidence of profound tissue reorganization in the urethra after SCI. We found signs of epithelial damage and recovery, accompanied by IUS atrophy, and marked sensory and sympathetic denervation. These changes likely contribute to the urinary impairment that follows SCI. This should be confirmed in future studies aiming to further unravel the pathophysiological mechanism of SCI-induced urinary dysfunction, paving the way for therapies exclusively targeting the urethra.

## Figures and Tables

**Table 1:** Urodynamic parameters from intact and SCI rats. Cystometric values of frequency, peak pressure, and amplitude of voiding contractions of spinal intact and SCI animals (1 and 4 weeks post-SCI). Cystometrograms were analyzed using the LabScribe software. Only contractions that reached at least 10 cm H_2_O of amplitude were considered for quantification. Data are represented as the mean ± standard deviation (one-way ANOVA followed by Turkey’s multiple comparison test; ** *p* < 0.01 compared to intact; #### *p* < 0.0001, ## *p* < 0.01 compared to 1w SCI).

	Spinal Intact	SCI—1 Week	SCI—4 Weeks
**Frequency**	0.60 ± 0.18	0.10 ± 0.08 ** *p* < 0.01	1.14 ± 0.15 ** *p* < 0.01 #### *p* < 0.0001
**Peak pressure**	44.27 ± 6.34	29.77 ± 1.29 ** *p* < 0.01	46.27 ± 4.06 ## *p* < 0.01
**Amplitude**	28.31 ± 10.63	3.29 ± 0.68 ** *p* < 0.01	23.65 ± 5.62 ## *p* < 0.01

**Table 2:** This table depicts the list of primary antibodies used in the present study. The source, host species, and dilution are indicated.

Primary Target	Dilution	Host Species	Manufacturer	Reference
SMA	1:1000	rabbit	Abcam	Ab124964
B-III tubulin	1:1000	rabbit	Synaptic systems	Sysy302302
GAP43	1:500	sheep	Novus	NBP1-41123
CGRP	1:1000	rabbit	Cell signaling	14959
VachT	1:1000	rabbit	Synaptic systems	Sysy139103
TH	1:500	rabbit	Abcam	Ab137869

**Table 3:** This table depicts the list of secondary antibodies used in the present study. The source, host species, and dilution are indicated.

Secondary Target	Dilution	Host Species	Manufacturer	Reference
Rabbit/Alexa 488	1:1000	donkey	ThermoFisher	Ab21206
Rabbit/biotinylated	1:200	swine	Dako Denmark	E0353
Sheep/Alexa 568	1:1000	goat	ThermoFisher	A21206
